# Predicting Deep Body Temperature (T_b_) from Forehead Skin Temperature: T_b_ or Not T_b_?

**DOI:** 10.3390/s22030826

**Published:** 2022-01-22

**Authors:** Jason T. Fisher, Urša Ciuha, Michael J. Tipton, Leonidas G. Ioannou, Igor B. Mekjavic

**Affiliations:** 1 Department of Automation, Biocybernetics and Robotics, Jozef Stefan Institute, Jamova 39, SI-1000 Ljubljana, Slovenia; jason.fisher@ijs.si (J.T.F.); ursa.ciuha@ijs.si (U.C.); ioannoulg@gmail.com (L.G.I.); 2 International Postgraduate School Jozef Stefan, Jamova 39, SI-1000 Ljubljana, Slovenia; 3 School of Sport, Health and Exercise Science, University of Portsmouth, Portsmouth PO1 2EF, UK; michael.tipton@port.ac.uk

**Keywords:** deep body temperature, skin temperature, heat strain, heatwave, contact thermography

## Abstract

There is a need to rapidly screen individuals for heat strain and fever using skin temperature (T_sk_) as an index of deep body temperature (T_b_). This study’s aim was to assess whether T_sk_ could serve as an accurate and valid index of T_b_ during a simulated heatwave. Seven participants maintained a continuous schedule over 9-days, in 3-day parts; pre-/post-HW (25.4 °C), simulated-HW (35.4 °C). Contact thermistors measured T_sk_ (T_forehead_, T_finger_); radio pills measured gastrointestinal temperature (T_gi_). Proximal-distal temperature gradients (ΔT_forehead–finger_) were also measured. Measurements were grouped into ambient conditions: 22, 25, and 35 °C. T_gi_ and T_forehead_ only displayed a significant relationship in 22 °C (r: 0.591; *p* < 0.001) and 25 °C (r: 0.408; *p* < 0.001) conditions. A linear regression of all conditions identified T_forehead_ and ΔT_forehead–finger_ as significant predictors of T_gi_ (r^2^: 0.588; F: 125.771; *p* < 0.001), producing a root mean square error of 0.26 °C. Additional residual analysis identified T_forehead_ to be responsible for a plateau in T_gi_ prediction above 37 °C. Contact T_forehead_ was shown to be a statistically suitable indicator of T_gi_ in non-HW conditions; however, an error of ~1 °C makes this physiologically redundant. The measurement of multiple sites may improve T_b_ prediction, though it is still physiologically unsuitable, especially at higher ambient temperatures.

## 1. Introduction

Two principal methods have been proposed to predict deep body temperature (T_b_) from the measurement of heat loss from the skin surface. One method measures the conductive heat loss pathway [1] and requires sensor contact with the skin surface. The second is a non-contact method, monitoring radiative heat loss with infrared thermography. Common to both methods are their inaccuracy in estimating absolute T_b_. Mekjavic and Tipton [2] concluded the prediction of T_b_ from one skin region, namely the forehead, is inaccurate, resulting in false positives and negatives. They suggest that other facial sites, such as the inner canthus of the eye, may prove superior to forehead skin temperature (T_sk_). They also recommend that T_sk_ gradients between proximal and distal sites, such as the forehead (proximal site) and fingertip (distal site), may provide an improvement in the prediction of T_b_. Namely, the proximal–distal skin temperature gradient (ΔT_sk_P-D) reflects perfusion of distal sites and may indicate whether the elevated temperature is due to heat strain or fever, the former causing peripheral vasodilatation, and the latter vasoconstriction.

Recently, the need to rapidly screen individuals using T_b_ prediction in industry has become more important for a number of reasons. Disregard for the control of greenhouse gases has resulted in global warming, with potentially devastating consequences for future generations. Among these consequences are summer heatwaves (HWs), originally infrequent and occurring only during the peak summer months, they are now increasing in frequency, magnitude, and duration [3]. In an industrial environment, HWs may affect the health and well-being of workers [4] and result in reduced labor productivity [5,6,7] as a result of occupational heat strain. It has been suggested that HWs may have a cumulative effect on workers, resulting in a residual effect several days after the ambient temperature returns to normal [5]. To try and mitigate the debilitating effects of HWs in the working environment, many countermeasures are available to reduce metabolic heat production and enhance heat loss, if only in the short term. The countermeasures include the availability of cold drinking water, cool and ventilated rooms during rest breaks, and cooling vests [7]. However, the possibility of monitoring workers for impending signs of heat strain, such as monitoring T_b_, has largely been ignored; a system of reactive rather than preventative monitoring is more common. 

Additionally, the recent pandemic of the Severe Acute Respiratory Syndrome Coronavirus 2 (SARs-CoV-2), resulting in a global coronavirus disease starting in 2019 (COVID-19), caused a lockdown of industrial activity during peaks of the COVID-19 waves in 2020. The manufacturing industry maintained some operations and has consequently taken the recommended precautions (i.e., masks, distancing, etc.) to safeguard the workforce. Some companies have implemented the monitoring of workers’ surface temperatures using infrared thermography (IRT) to estimate T_b_. Those identified by the scanners as having elevated body temperature, for whatever reason, are not allowed entry. 

In view of increasing reliance on the prediction of T_b_ from T_sk_, the present study evaluated whether contact measurements of T_sk_ can provide a suitable surrogate for direct measurement of T_b_; for the purpose of screening workers for SARs-CoV-2 virus infection and impending heat strain during summer HW. It was hypothesized that T_sk_ would produce a significant association with T_b_, but measurement of more sites to generate a ΔT_sk_P-D will produce a stronger association, as hypothesized by Mekjavic and Tipton [2].

## 2. Materials and Methods

This study was part of a program of research conducted within the framework of the European Commission Heat Shield project, investigating the effect of HWs on the health, well-being, and labour productivity of workers in five key European industries (manufacturing, agriculture, construction, logistics, tourism). During four previous HWs, conditions within an industrial manufacturing plant employing 1500 workers (odelo d.o.o., Prebold, Slovenia) were monitored [5]. Due to the difficulty of continuous 24-h physiological monitoring of workers during a HW, a study was conducted simulating the industrial process in controlled laboratory conditions [8], using data from the HWs measured in central Slovenia. Consequently, measurements of T_sk_ and T_b_ were conducted hourly throughout a 9-day study, including both normothermic and simulated HW conditions, to assess the association with T_b_ using indirect measurements. 

### 2.1. Participants

A sample size of seven participants was deemed to provide sufficient power to detect a statistical significance, assuming an α of 0.001 and β of 0.99 (G*Power Version 3.1.9.6, Bonn, Germany) using an effect size of effect size (d) of 1.8834 (f = 0.9417), based on the results of a previous study [8,9]. Seven young, healthy males (mean (SD); age: 21.1 (1.1) years; body stature: 180 (6.1) cm; body mass: 81.5 (15.6) kg; body mass index: 25.1 (4.4) kg·m^2^) participated in the study, which had received prior approval (Approval no. 0120-402/2020/4: 20 October 2020) by the Committee for Medical Ethics at the Ministry of Health (Republic of Slovenia). All were non-smokers, engaged in regular physical activity recreationally, and were free from known cardiovascular, respiratory, and autonomic disease. Prior to the commencement of the study, the participants were informed of the details of the experimental protocol and were familiarized with the procedures, before signing an informed consent agreement. The participants were aware that they could terminate participation in the study at any time during the 10-day duration.

### 2.2. Protocol

The study was conducted at the PlanHab facility (European Space Agency ground-based research facility) at the Olympic Sports Centre Planica (Rateče, Slovenia). Participants were confined to the facility for 9-days and had access to their rooms, a common area, laboratory, and dining area. They were provided with three meals and two snacks each day (breakfast, lunch, afternoon snack, dinner, evening snack) and could drink water ad libitum.

On arrival at the facility, the participants were acquainted with the entire facility and were familiarized with all the experimental procedures. They were instructed to refrain from venturing outside the designated areas of the facility, as the temperature and humidity were regulated only in the designated areas, using heaters controlled by temperature regulators. Ambient humidity within the laboratory remained constant at ~45%. The protocol was designed to mimic the routine daily activities in a manufacturing plant, as well as some of the activities at home. Participants were awakened each day at 0700 hrs. After breakfast, they entered the laboratory at 0840 hrs, which was arranged as a series of workstations equipped with personal computers. The work shift lasted until 1800 with breaks for snacks and lunch. Upon completion of the work shift, participants had dinner and then retired to their common area or individual rooms. Lights out was at 2300 hrs. This was the daily routine for nine consecutive days.

During the 9-day confinement, the temperatures within the living quarters and workplace (i.e., laboratory) were regulated, as displayed in Table 1. The first 3 days (pre-HW) represented normal conditions. The simulated HW was initiated at midnight at the end of day 3, with temperatures increasing in all areas. At midnight on day 6, the night-time/daytime temperature profile was re-adjusted to the same profile as in the first 3 days (post-HW). Experiments took place in ambient conditions of a 19.8 ± 1.8 Wet-Bulb Globe Temperature (from www.wunderground.com; accessed on 14 January 2022).

### 2.3. Measurements

Each morning the participants ingested a calibrated telemetric radio pill (Body Cap, Caen, France), a thermistor was secured to their forehead (T_forehead_), and a distal phalanx pad was attached to the middle finger (T_finger_) (iButton, Type DS1921H, Maxim/Dallas Semiconductor Corp., Dallas, TX, USA). These devices provided continuous measurement of gastrointestinal temperature (T_gi_) and T_sk_, respectively, on each day. Validation of the calibrated telemetric radio pill against rectal thermistor during rest, water immersion, and steady-state exercise revealed no significant differences; furthermore, the system produces effective validity and test-retest reliability [10,11]. Additionally, the validation of iButton thermistors against calibrated thermocouples revealed no significant difference during steady-state, though response time to changes in temperature was slower than thermocouples [12].

### 2.4. Analyses

T_gi_ and T_sk_ were measured continuously, and an average of the last 10 min was taken in each hour for 23-h, every day. This averaging period was chosen to avoid potential artefacts by using a stable 10-min period. Each day, telemetric pills were ingested at 0700 hrs, immediately after waking up, and T_sk_ iButtons were attached to the skin in the evening at 2230 hrs. Temperature measurements were recorded during three distinct ambient conditions: 22 °C, 25 °C, and 35 °C. ΔT_sk_P-D, an index of blood flow [13], was calculated between the forehead and fingertip (ΔT_forehead–finger_). When measured at the forearm–finger or calf–toe, a value ≥2 °C represents vasoconstriction and ≤0 °C represents vasodilation [14,15]. In the present study, in which the ΔT_sk_P-D was assessed from T_sk_ at the forehead and fingertip, the thresholds for vasoconstriction and vasodilatation may likely be dissimilar to those reported by previous studies using the forearm–fingertip skin temperature gradient as an index of perfusion. Holm, et al. [16] have previously investigated the use of the forehead–fingertip skin temperature gradient as an index of mortality in hospital patients. 

Means, standard deviations, and coefficient of variation (CoV) were calculated for T_gi_, T_forehead_, and T_finger_ (Table 2).

The data, following calculation of normality by a Shapiro–Wilk test, were assessed using either a Pearson’s Correlation Coefficient or a Spearman’s Rank Correlation Coefficient. Additionally, a multiple linear regression using T_forehead_, T_finger_ and ΔT_forehead-finger_ was conducted. All statistical tests were completed using an alpha value of *p* < 0.05 and conducted using IBM SPSS Statistics (Version 26, Armonk, NY, USA).

In addition to the multiple linear regression, root mean square error (RMSE) was also calculated between measured T_gi_ and predicted T_gi_ as produced from a regression equation, using the following equation [17]:

(1) $$\mathrm{RMSE}={\left[\overset{N}{\underset{i=1}{\sum }}\frac{{\left(Z_{f\left(i\right)}-Z_{O\left(i\right)}\right)}^{2}}{N}\right]}^{1/2}$$

where,

Z_f_ = forecast value

Z_o_ = observed value 

N = sample size 

## 3. Results

All participants completed the 9-day confinement. There were no untoward effects of the 3-day HW. The physiological responses and labor productivity during the simulated normal weather and HW periods have been presented elsewhere [8].

### 3.1. Relationship between T_sk_ and T_gi_

To assess the true relationship between T_forehead_ and T_gi_, measurements from every day were compared simultaneously, encompassing all ambient conditions. The range of temperatures observed was greater for T_forehead_ (32.2–36 °C) than for T_gi_ (36.1–37.7 °C), whereas the average temperature of all measurements was higher for T_gi_ (T_gi_: 36.9 ± 0.4 °C; T_forehead_: 33.9 ± 1.4 °C), a significant difference (*p* < 0.001). A significant relationship was identified between the measurements of T_forehead_ and T_gi_ (r = 0.653; *p* < 0.001). 

### 3.2. T_sk_ and T_gi_ at Different Ambient Temperatures (HW vs. Non-HW)

**Figure 1:**
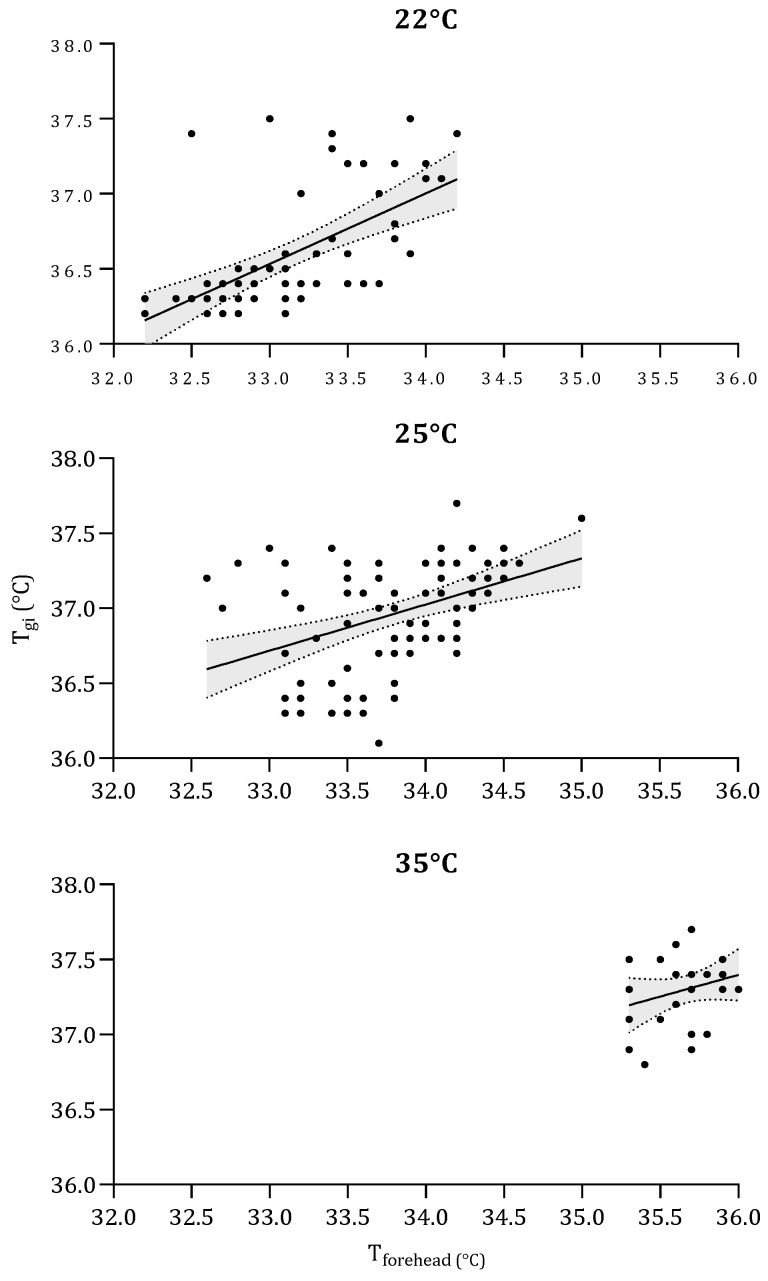
The relation between the temperature of the gastrointestinal tract measured with a radio pill (T_gi_) and the temperature of the forehead using a contact thermistor (T_forehead_). Measurements were obtained while participants were exposed to three ambient temperatures: 22 °C (upper panel), 25 °C (middle panel), and 35 °C (lower panel). Regression lines with associated 95% confidence bands for each temperature are also shown.

The above correlation analysis of the relationship between T_forehead_ and T_gi_ was repeated for the individual HW (35 °C) and non-HW (22 °C and 25 °C) ambient temperatures, as shown in Figure 1. A significant relationship was observed for the 22 °C (r = 0.591; *p* < 0.001) and 25 °C (r = 0.408; *p* < 0.001) ambient conditions, whereas there was no significant relationship at 35 °C (r = 0.263; *p* < 0.185). Table 2 displays mean (SD) T_sk_ and T_gi_ values measured in each ambient condition.

### 3.3. Proximal-Distal Temperature Gradient Prediction

Mekjavic and Tipton [2] suggest that an index derived from measurements made at multiple sites might provide a more accurate temperature screening, primarily using areas where the skin is exposed (i.e., face and hands). When creating a T_sk_P-D between the forehead and fingertip (ΔT_forehead–finger_), the correlation between this variable and T_gi_ was significant (r = 0.637; *p* < 0.001). Additionally, a multiple linear regression for prediction of T_gi_ using T_forehead_, T_finger_, and ΔT_forehead–finger_ produced a significant linear model using T_forehead_ and ΔT_forehead–finger_ only (r^2^ = 0.588; F: 125.771; *p* < 0.001):

(2) $${\mathrm{Predicted}\;T}_{\mathrm{gi}}=29.349+\left(0.225\times T_{\mathrm{forehead}}\right)+\left(0.154\times ∆T_{\mathrm{forehead}-\mathrm{finger}}\right)\;$$

**Figure 2:**
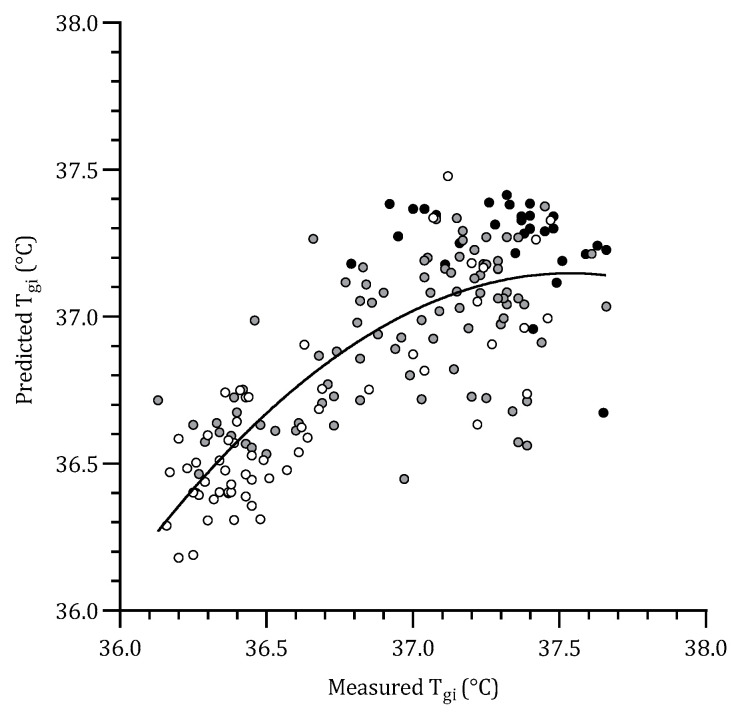
Relationship between measured temperature of the gastrointestinal tract (T_gi_) and predicted T_gi_ using Equation (1). Measurements and predictions based on skin temperatures were obtained while participants were exposed to three ambient temperatures: 22 °C (white dots), 25 °C (grey dots), and 35 °C (black dots). A second-order polynomial trendline (y = −0.4464x^2^ + 33.512x − 591.75) represents the best fit (r^2^ = 0.63).

This linear regression model describes a suitable fit between the measured and predicted values of T_gi_. RMSE analysis of this regression equation established an error of 0.26 °C between the actual and predicted T_gi_. Figure 2 displays the correlation between the measured and predicted T_gi_, which exhibits a plateau at higher measured T_gi_. A second-order polynomial trendline was chosen (solid line in Figure 2) to best represent the associated fit of the correlation (r^2^ = 0.63).

## 4. Discussion

Screening workers for elevated T_b_ has become of particular importance with the prevalence of two major global maladies, global warming and the COVID-19 pandemic. Both of which cause dangerous elevations in T_b_ and have potentially serious, if not fatal, consequences. Presently, workers in the industry are being screened primarily for elevations in T_b_ arising from a viral infection. However, in the future, any such valid methodology has the potential to be used for monitoring workers for heat strain, particularly during episodes of summer HWs. The assessment of the currently used approach for screening for elevated T_b_ was the aim of the present study. The principal finding was that neither single skin sites (i.e., hand, forehead), nor the T_sk_P-D in combination with T_forehead_, were able to provide a physiologically accurate index of T_b_ (i.e., gastrointestinal temperature). The methodological approach of predicting T_b_ from T_forehead_ is therefore not valid. 

### 4.1. Prediction of T_b_ Using Measurements of T_forehead_ and ∆T_forehead–fingertip_

The statistical analysis in Section 3.1 revealed a significant correlation between the T_forehead_ and T_gi_, but the association with absolute T_b_ on this basis may vary by as much as 2 °C. Therefore, based on statistical analysis, T_forehead_ appears to be a suitable index of T_b_, however, this correlation is of limited physiological relevance as it may generate false positive/negative values. Of particular concern is the fact that the correlation becomes statistically non-significant during simulated HW conditions, conditions where an accurate prediction in an industrial setting would be required. The present study used contact thermometry to measure skin temperature, the method of choice in industry being T_forehead_, obtained with infrared thermography (IRT). Using this technology, the measurement of surface T_sk_ may be adequate; however, as demonstrated by the results of the present study, the subsequent derivation of T_gi_ from the measurement of T_sk_ at one site, the preferred site being the forehead, is not physiologically valid. 

The recent proposal of Mekjavic and Tipton [2], which suggests additional sites to that of the forehead alone might provide a better outcome in the prediction of T_gi_, was also evaluated by conducting a linear regression to calculate T_gi_ with the proximal-distal skin temperature gradient (ΔT_forehead–finger_), and skin temperatures. This regression proved statistically significant, resulting in smaller errors in the predictions of T_b_. Furthermore, a polynomial curve fit the relationship between measured and predicted T_gi_ identified a plateau at higher levels of predicted T_gi_ (Figure 2). This suggests that the association appears to be accurate at lower temperatures; however, it begins to underestimate T_b_ as T_gi_ increases. Residual analysis of independent variables in the regression equation identifies T_forehead_ as a contributor to this plateau due to increased variability and thus error at higher ambient temperatures. Additionally, whilst the average T_gi_ in the HW conditions was 37.3 °C, T_forehead_ only reached 35.5 °C, which means it was incapable of linearly matching rises in T_gi_ during higher ambient conditions. The combination of these two sources of error likely caused the plateau in the relationship between measured and predicted T_gi_, making it unsuitable to use T_forehead_ as a prediction tool. It should also be emphasized that the industrial tasks simulated in the present study were that of checking the functioning of circuit boards; thus, a seated task. Any method for predicting heat strain in an industrial environment will need to be validated with tasks requiring elevated endogenous heat production, further increasing T_gi_ above T_forehead_.

The ΔT_sk_P-D between the forearm and fingertip has been demonstrated as an appropriate index of the perfusion of the fingers [13,14,15]. During exposure to a hot environment, as in the present study, a high distal (fingertip) T_sk_ would reflect vasodilatation, thus activation of the thermoregulatory heat loss mechanism. We hypothesized that if T_forehead_ was a valid surrogate of T_b_, when combined with an index of peripheral perfusion, such as ΔT_sk_P-D, this could provide an index of heat strain. However, unlike T_gi_, T_forehead_ varied with ambient temperature, such that the observed variations in T_gi_ of ±1.5 °C, were accompanied by variations in T_forehead_ of ±3.8 °C, casting doubt on the validity of T_forehead_ as a valid surrogate measurement of T_b_. Nevertheless, the ΔT_forehead–finger_ alone displayed a significant relationship with T_gi_. Furthermore, a multiple regression combining ΔT_sk_P-D with T_forehead_ generated a regression equation, with an improved association with T_gi_. The physiological validity of the derived regression model should be evaluated with a separate group of female and male subjects, of different ages, under conditions of elevated ambient temperatures, as would be experienced in the industry and during HWs. 

### 4.2. Effect of Ambient Temperature on the Relation between Tsk and Tb

Mass screening of workers for elevated T_gi_ in an industrial setting may help to protect against heat stress or avoid the spread of viral disease. The ambient temperatures at which these measurements are taken may vary depending on the location of the measurement (indoor vs. outdoor), time of day (day shift vs. night shift), weather, and season. The large variation in T_sk_, with little change in T_gi_, is of concern with regard to the association of T_b_ with T_sk_. In the present study, measurements taken in normal temperature (22 and 25 °C) ambient conditions provide a statistically significant relationship with T_gi_, whereas measurements conducted during simulated HW (35 °C) conditions provided no statistically significant relationship. In the present study, increases in T_b_ were the result of high ambient temperatures. In contrast, a febrile temperature is the result of elevated endogenous heat production combined with decreased heat loss (vasoconstriction). Any method proclaiming to be able to predict T_b_ of active and/or febrile individuals regardless of the ambient temperature should be appropriately validated. Manufacturers of currently available scanners based on IRT technology do not provide the algorithms used to predict T_b_ based on T_forehead_, nor do they provide any information regarding the validation of such algorithms. Due to the proven global importance of screening individuals for elevated T_b_, it should only be a matter of time before this is regulated. 

### 4.3. Accuracy of IRT to Contact Thermography

The aim of the present study was to assess the association of T_sk_ with T_b_ using contact thermography and not to validate IRT as a method for predicting T_b_. However, IRT is the most commonly used method of measuring skin temperature in applied settings such as workplaces and hospitals, and its validity and accuracy should be considered in future T_sk_ predictions. The validity of IRT as a measurement of T_sk_ has been heavily debated, particularly with reference to its overestimation and comparison to a ‘gold standard’ of T_sk_ measurement. Maley et al. [18] propose that during hand rewarming, following cold water immersion, IRT overestimates T_sk_ measured by contact thermometry by 1.80 °C. However, this was countered by Havenith and Lloyd [19], who suggest that methodological issues such as camera accuracy and calibration commonly occur, and that contact thermometry cannot be considered a ‘gold standard’.

Any system for mass screening of workers based on the prediction of body temperature from forehead T_sk_ derived with IRT would need to utilize an infrared camera of high accuracy as differences occur commonly. Ng et al. [20] reported significant differences among the three infrared scanners used to measure T_forehead_. The differences among these scanners were as high as ±2 °C. Such discrepancies among infrared cameras are also reflected in their ability to accurately measure T_sk_ when compared to contact thermography. Although a strong correlation between contact thermometry and non-contact IRT thermography has been reported [18,21], the authors reported that T_sk_ measured with IRT was 2.3 °C lower than that measured with a thermistor [21]. The above comparisons were made during a sleep study [21] and at rest [18]. During dynamic movement and exercise, as would be anticipated in an industrial setting, the agreement between contact and IRT measurements of T_sk_ is poor [22,23]. Irrespective of the validity achieved by IRT, the type of device specifications stipulated by the ‘Journal Temperature Toolbox’ [24], may be too stringent and impractical for many workplaces.

### 4.4. Prediction of Deep Body Temperature

Infrared scanners providing a predicted value of T_b_ based on a measurement of T_sk_ at a single site do so using proprietary algorithms, which are not available for scrutiny. This is unsatisfactory and unacceptable considering the impact elevated body temperature, whether due to viral infection or summer HW, has had not only on the industry but all aspects of our lives globally. The present study illustrates the errors in the association of T_b_ with T_sk_ that occur under controlled laboratory conditions, in which the measurements were conducted by trained individuals. It also emphasizes the need to discern between statistical and physiological significance. As an example, the correlation between T_forehead_ and T_gi_ (Figure 1) may be statistically significant, indicating that an increase in one variable is observed as an increase in the other; this relation does not, however, provide an accurate assessment of T_b_. Alternatively, using a regression equation of multiple measurement sites provided a significant prediction of T_gi_, the physiological significance of which is made clear using RMSE. This analysis of the regression equation proposes that the error between actual and predicted T_gi_ is as low as 0.3 °C, enabling more accurate extrapolation of T_gi_ from T_sk_ to occur. For measurements of T_b_, the difference in values at one site could be the difference between a healthy temperature and heat strain or fever. It is most likely that future strategies of predicting T_b_ from exposed T_sk_ may need to incorporate several sites, and not just one, as suggested by Mekjavic and Tipton [2], as demonstrated in the present study for assessment of heat strain in workers during HW.

### 4.5. Limitations

As detailed above, differences lie in the mechanisms relating to changes in T_b_, leading to differential heating and perfusion responses during either ambient heating or fever. The present study produced an equation for the prediction of T_b_ using several sites when participants were experiencing ambient heating at rest. Additional testing should consider the T_sk_ and T_b_ responses to the unique aspects of fever and exercise as methods of heating the human body. In addition, the participants in the present study, young, healthy males, did not appear to experience undue heat strain based on their T_gi_. Though these participants were exposed to the conditions of a previously recorded HW [5], suggesting other non-thermal factors such as morphology, gender, acclimation, etc., should be considered in the prediction algorithm produced. Due to the relatively small and homogenous sample, the results of the present study should only be used as an example of the type of error associated with T_sk_ prediction. Finally, while the study design reflected certain applied conditions such as working schedules and tasks, the external validity should be cautioned and additional research with larger sample sizes in applied conditions advised.

## 5. Conclusions

Measurement of contact T_sk_ at the forehead appears to be a suitable site from which T_gi_ can be extrapolated at lower ambient temperatures. However, while statistically significant, this relationship cannot be considered physiologically appropriate due to an error of ~1 °C. The measurement of multiple sites, including a proximal-distal temperature gradient, may provide a more suitable prediction of T_b_ with a lower error (0.3 °C), however again this is not appropriate due to a plateauing of the prediction efficacy at higher temperatures, likely due to lower and more variable T_sk_ measurements. The methodological approach of predicting T_b_ from T_sk_ is therefore not physiologically valid in young males, particularly in higher ambient temperatures. In the future, indirect T_sk_ measurements should consider the effect of ambient temperature, the use of multiple sites, inclusion of a perfusion index, and the source of raised T_b,_ in their algorithms.

## Figures and Tables

**Table 1:** Temperature during daily work and rest periods. Temperature is presented as a mean (SD) of each 3-day testing condition.

	Work (0840–1800 hrs)	Rest/Sleep (1800–0840 hrs)
	Temperature (°C)	Temperature (°C)
Pre-HW	25.4 (0.3)	22.3 (0.5)
HW	35.5 (0.3)	26.3 (0.8)
Post-HW	25.5 (0.7)	23.1 (0.7)

HW: Heatwave. Pre-HW: Testing days 1–3. HW: Testing days 4–6. Post-HW: Testing days 7–9.

**Table 2:** Mean (±SD), and coefficient of variation (CoV) of Tsk and Tgi measurements at each ambient condition.

Ambient Condition	Measurement	Mean (SD)	CoV (%)
22 °C	T_gi_	36.7 (0.4)	1.2
	T_forehead_	34.2 (1.4)	4.1
	T_finger_	33.2 (0.5)	1.5
25 °C	T_gi_	37.0 (0.4)	1.0
	T_forehead_	33.9 (1.3)	3.7
	T_finger_	33.8 (0.5)	1.4
35 °C	T_gi_	37.3 ± 0.2	0.6
	T_forehead_	35.9 ± 0.7	1.9
	T_finger_	35.5 ± 0.6	1.7

T_gi_: gastrointestinal temperature. T_forehead_: forehead temperature. T_fingertip_: fingertip temperature.

## Data Availability

Data available on request.
